# A Clinical-Radiomics Nomogram Based on the Apparent Diffusion Coefficient (ADC) for Individualized Prediction of the Risk of Early Relapse in Advanced Sinonasal Squamous Cell Carcinoma: A 2-Year Follow-Up Study

**DOI:** 10.3389/fonc.2022.870935

**Published:** 2022-05-16

**Authors:** Naier Lin, Sihui Yu, Mengyan Lin, Yiqian Shi, Wei Chen, Zhipeng Xia, Yushu Cheng, Yan Sha

**Affiliations:** Department of Radiology, Eye & ENT Hospital, Shanghai Medical College, Fudan University, Shanghai, China

**Keywords:** sinonasal cancer, recurrence, apparent diffusion coefficient, radiomics, nomogram

## Abstract

**Purpose:**

To develop and validate a nomogram model combining radiomic features and clinical characteristics to preoperatively predict the risk of early relapse (ER) in advanced sinonasal squamous cell carcinomas (SNSCCs).

**Methods:**

A total of 152 SNSCC patients (clinical stage III-IV) who underwent diffusion-weighted imaging (DWI) were included in this study. The training cohort included 106 patients assessed at the headquarters of our hospital using MR scanner 1. The testing cohort included 46 patients assessed at the branch of our hospital using MR scanner 2. Least absolute shrinkage and selection operator (LASSO) regression was applied for feature selection and radiomic signature (radscore) construction. Multivariable logistic regression analysis was applied to identify independent predictors. The performance of the model was evaluated using the area under the receiver operating characteristic curve (AUC), calibration curve and decision curve analysis (DCA). Furthermore, the patients were classified into high- or low-risk ER subgroups according to the optimal cutoff value of the nomogram using X-tile. The recurrence-free survival probability (RFS) of each subgroup was assessed.

**Results:**

ER was noted in 69 patients. The radscore included 8 selected radiomic features. The radscore, T stage and surgical margin were independent predictors. The nomogram showed better performance (AUC = 0.92) than either the radscore or the clinical factors in the training cohort (*P* < 0.050). In the testing cohort, the nomogram showed better performance (AUC = 0.92) than the clinical factors (*P* = 0.016) and tended to show better performance than the radscore (*P* = 0.177). The nomogram demonstrated good calibration and clinical utility. Kaplan-Meier analysis showed that the 2-year RFS rate for low-risk patients was significantly greater than that for high-risk patients in both the training and testing cohorts (*P* < 0.001).

**Conclusions:**

The ADC-based radiomic nomogram model is potentially useful in predicting the risk of ER in advanced SNSCCs.

## Introduction

Malignancies involving the sinonasal tract are uncommon, accounting for approximately 3%–5% of all head and neck malignancies (1). Among the histological varieties of malignancies, the most common primary cancer is sinonasal squamous cell carcinoma (SNSCC) (2). SNSCC typically presents with nonspecific symptoms at an advanced stage with involvement of adjacent structures such as the infratemporal fossa, skull base and orbit (3), potentially resulting in the incomplete resection of the whole tumor and positive surgical margins. Thus, a high frequency of local failure and recurrence is observed (4).

Tumor-node-metastasis (TNM) staging is one of the most important prognostic factors guiding the treatment options for SNSCC patients. However, due to highly heterogeneous tumor biology, clinical outcomes may be completely different even in patients with the same stage of disease. To date, the identification of more reliable markers to facilitate individualized prediction of the risk of early relapse (ER), particularly in advanced SNSCC, is urgently needed.

Over the last few years, radiomics has become a research hotspot. It allows the extraction of a large number of image features of the total tumor, which can highlight the heterogeneity and characteristics of the tumor by acting as a whole tumor virtual biopsy. Recently, a few studies revealed that radiomics based on images combined with clinical factors could aid in improving the accuracy of recurrence prediction in several cancers, such as nasopharyngeal carcinoma (5), gastric cancer (6) and hepatocellular carcinoma (7). However, in these studies, the features were all extracted from computed tomography (CT) images, the soft tissue resolution of which was lower than that of MRI. To date, of the available studies, only a few studies (8–10) have focused on the application of radiomics in sinonasal tumors, and most of them have exclusively focused on its use for differential diagnosis.

Diffusion weighted imaging (DWI) can reflect the random movement of molecules of water at the cellular level. With better characterization of tissues, apparent diffusion coefficient (ADC) values calculated from DWI have been increasingly used in sinonasal lesions and shown to be a promising biomarker to discriminate benign from malignant tumors as well as to identify different histopathological types of sinonasal malignancies (11). However, at present, the usefulness of a clinical-radiomics nomogram based on ADC images for predicting the recurrence and survival state in advanced SNSCC patients preoperatively has not been developed.

Thus, using different MR devices, the current study was conducted to explore whether an ADC-based nomogram combining the radiomic signature (radscore) with clinical factors can predict ER in patients with advanced SNSCC.

## Patients and Materials

### Patients

A total of 152 patients (115 male, 37 females; age range, 17-84 years; mean, 55.41±14.59 years) with histologically confirmed SNSCC who visited our hospital between December 2013 and October 2019 were enrolled. The SNSCC patients were at an advanced stage, i.e., American Joint Committee on Cancer (AJCC) stage (7^th^ edition) III-IV. All patients underwent surgical treatment with transnasal endoscopic resection or open surgical resection. A total of 145 patients (95.4%) were treated with radiotherapy, including the 3D conformal radiotherapy (3D-CRT) technique or intensity-modulated radiotherapy (IMRI). Adjuvant chemotherapy prior to or after surgery was performed in 31 patients (20.4%). The patients were divided into two cohorts. The training cohort included patients assessed in the headquarters of our hospital using MR scanner 1, and the independent external testing cohort consisted of patients assessed at a branch of our hospital using MR scanner 2. The follow-up time in all patients was 24 months. The patients who experienced relapse within 2 years were defined as the ER cohort, whereas patients who did not experience relapse within 2 years were classified as the nonearly recurrence (NER) cohort. Recurrence-free survival probability (RFS) was calculated from the day after treatment to the date of relapse, death from any cause, or last follow-up (24 months). The Institutional Review Board of our hospital approved this retrospective study, and informed consent was obtained from all patients.

### Image Acquisition, Segmentation and Feature Extraction

Preoperative sinonasal MRI scans including axial DWI within half a month prior to the operation. MR scanner 1 was applied in the training cohort (Magnetom Verio; 3.0 T, Siemens Healthcare, Erlangen, Germany) with a 12-channel head and neck coil, and MR scanner 2 was applied in the testing cohort (Magnetom Prisma; 3.0 T, Siemens Healthcare, Erlangen, Germany) with a 64-channel head and neck coil. DWI was performed using the readout-segmented echo-planar diffusion weighted imaging (RESOLVE-DWI).This high-resolution DWI system can offer a higher image quality than conventional DWI and reduce the disturbance from surrounding bones and air. The parameters for DWI were as follows: TR/TE = 4700/66 ms (scanner 1), 3000/56 ms (scanner 2), b values = 0, 1000 s/mm^2^ (scanner 1 and scanner 2); thickness = 3 mm (scanner 1) and 5mm (scanner 2); number of segments = 5 and field of view (FOV) = 230 × 230 mm^2^ (scanner 1 and scanner 2). ADC maps were automatically derived from DWI images. The exclusion criteria were as follows: (a) patients who had received therapy before MRI examination; (b) missing information on clinicopathological variable and (c) insufficient lesion size or image quality for diagnosis.

**Figure 1:**
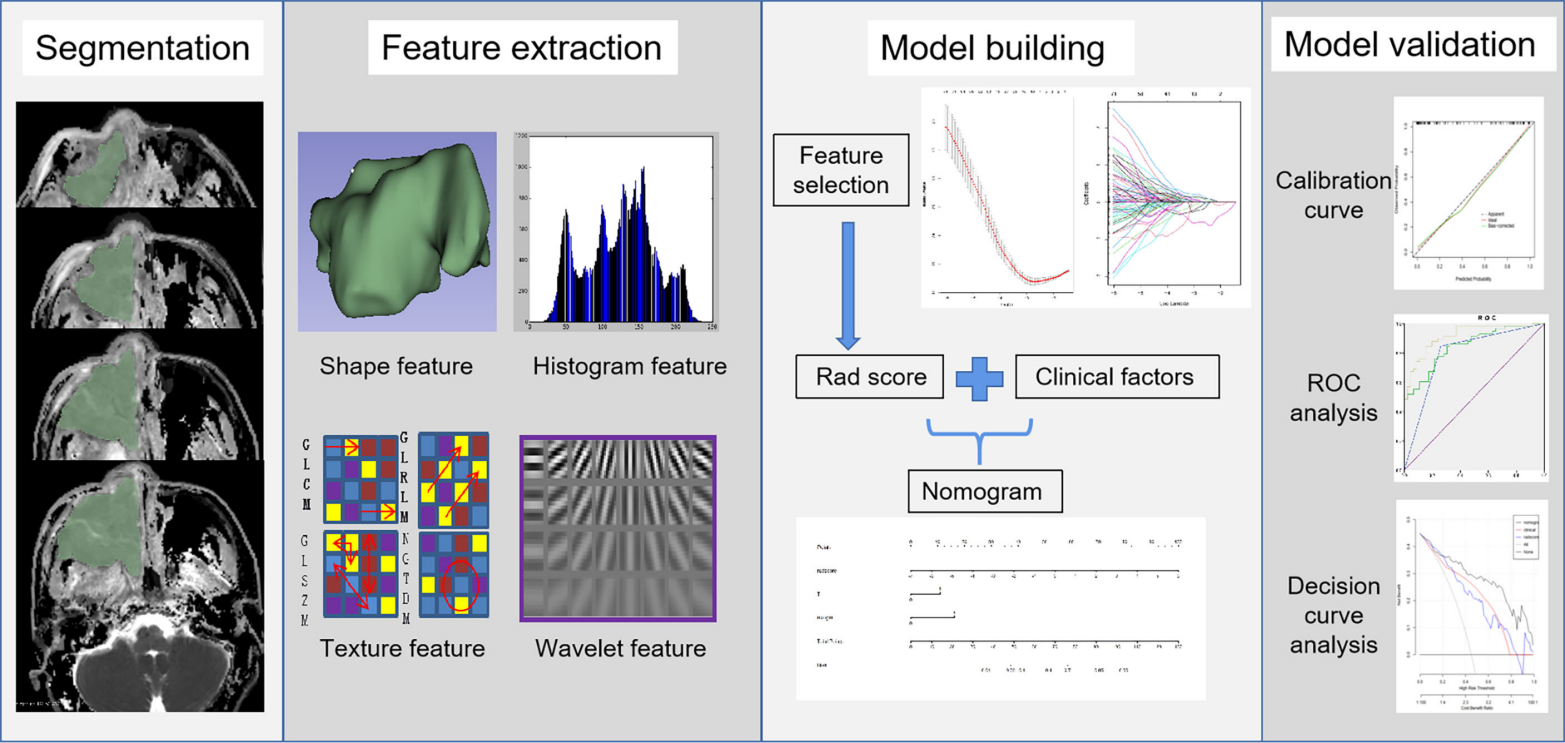
Flowchart of radiomics for predicting the risk of ER in this study.

The radiomics workflow is displayed in **Figure 1**.

Image segmentation was performed independently by 2 diagnostic radiologists with over 10 years of experience in radiology using the “Segment Editor” module of the software program 3D Slicer (version 4.8.1). The 3-dimensional regions of interest (ROIs) were outlined slice by slice on the ADC maps to cover the whole tumor with reference to T2WI and contrast-enhanced images avoiding the obvious necrosis and cystic areas within the tumor. Radiomic features were extracted with the “Radiomics” module and classified as (a) shape features; (b) first-order features; (c) texture features; and (d) wavelet-based features. The texture features included the gray level dependence matrix (GLDM), gray level cooccurrence matrix (GLCM), gray level run length matrix (GLRLM), gray level size zone matrix (GLSZM), and neighboring gray tone difference matrix (NGTDM). The inter-operator variability of the radiomic features was assessed with the intraclass correlation coefficient (ICC). Features with ICCs > 0.75 were included in subsequent analysis.

### Radiomics Feature Selection and Construction of the Radiomics Signature

All radiomic features were normalized (Z-score transformation) to improve the comparability of the data. Then, we used the least absolute shrinkage and selection operator (LASSO) logistic regression model and 7-fold cross-validation to select the most valuable features based on the training set and build the Radiomics Signature (radscore). A formula was generated using a linear combination of selected features that were weighted by respective coefficients. The radscore was computed according to the formula.

### Construction and Validation of the Nomogram

Multivariable logistic regression analysis was applied in the training group based on the following candidate factors: age, sex, smoking history, origin type, lateral location, maximum diameter, T stage, N stage, M stage, surgical margin and radiomic signature. Factors with P *<*0.050 were included in the nomogram as clinical predictors of tumor ER.

We compared the predictive performances of the radscore, clinical factors and nomogram model using the area under the curve (AUC) of the receiver operating characteristic curve (ROC). Then, the calibration curve accompanied by the Hosmer-Lemeshow test was used to evaluate the accuracy of the nomogram model. Decision curve analysis (DCA) of the nomogram was applied to summarize the clinical value.

Furthermore, the patients were classified into high- or low-risk ER subgroups according to the optimal cutoff value of the nomogram using X-tile. The RFSs of high- and low-risk ER subgroups were assessed in both the training and testing cohorts using Kaplan-Meier survival analysis.

### Statistical Analysis

SPSS (version 23.0), Medcal (version 19.0) and R software (version 4.0) were used to perform the statistical analysis. LASSO regression, nomogram generation, calibration curve calculation, and DCA were conducted with the R packages “glmnet”, “rms” and “dca.r” packages, respectively. Student’s *t test* and the Mann-Whitney U test were used to compare continuous variables with normal and abnormal distributions, respectively. Categorical variables were assessed using the chi-square (χ^2^) test.

Here, X-tile software was used (version 3.6.1) to determine the optimal cutoff value of the nomogram and to divide the patients into high- and low-risk subgroups. RFS was calculated using the Kaplan-Meier method. *P* value < 0.050 was considered statistically significant.

## Results 

In our study, there were 39 cases of AJCC stage III and 113 cases of stage IV (IVa: 75 cases, IVb: 31 cases, IVc: 7 cases). Sixty-nine patients relapsed within 2 years and local recurrence was the main reason among them. **Table 1** shows the characteristics of patients in both cohorts. No significant differences in the presence of ER (*P* = 0.755), patient age (*P* = 0.361), sex (*P* = 0.623), smoking rate (*P* = 0.703), lesion laterality (*P* = 0.633), maximum lesion diameter (*P* = 0.392 ), origin type (*P* = 0.118), T stage (*P* = 0.966), N stage (*P* = 0.317), M stage (*P* = 0.459) or surgical margin (*P* = 0.130) were noted between the two cohorts.

**Table 1:** Characteristics of patients in the training and testing cohorts.

Characteristic	Training Cohort (n = 106)			Testing Cohort (n = 46)		
	NER (n = 57)	ER (n = 49)	*P*	NER (n = 26)	ER (n = 20)	*P*
Age, No. (%)			0.131			0.917
≥55years old	34 (59.6%)	22 (44.9%)		16 (61.5%)	8 (40.0%)	
< 55 years old	23 (40.4%)	27 (55.1%)		10 (38.5%)	12 (60.0%)	
Gender			0.270			0.239
Female	17 (29.8%)	10 (20.4%)		4 (15.4%)	6 (30.0%)	
Male	40 (70.2%)	39 (79.6%)		22 (84.6%)	14 (70.0%)	
Smoking			0.210			0.088
Yes	21 (36.8%)	24 (49.0%)		13 (50.0%)	5 (25.0%)	
No	36 (63.2%)	25 (51.0%)		13 (50.0%)	15 (75.0%)	
Origin type			0.980			0.479
DN-SCC	42 (73.7%)	36 (73.5%)		17 (65.4%)	11 (55.0%)	
IP-SCC	15 (26.3%)	13 (26.5%)		9 (34.6%)	9 (45.0%)	
Lesion laterality			0.185			0.224
Unilateral	49 (86.0%)	46 (93.9%)		24 (92.3%)	16 (80.0%)	
Bilateral	8 (14.0%)	3 (6.1%)		2 (7.7%)	4 (20.0%)	
Maximum diameter			0.938			0.239
< 5cm	33 (57.9%)	28 (57.1%)		15 (57.7%)	8 (40.0%)	
≥ 5cm	24 (42.1%)	21 (42.9%)		11 (42.3%)	12 (60.0%)	
T Stage			0.002*			0.031*
1/2/3	22 (38.6%)	6 (12.2%)		10 (38.5%)	2 (10.0%)	
4a/4b	35 (61.4%)	43 (87.8%)		16 (61.5%)	18 (90.0%)	
N Stage			0.265			0.733
0	48 (84.2%)	37 (75.5%)		23 (88.5%)	17 (85.0%)	
1/2	9 (15.8%)	12 (24.5%)		3 (11.5%)	3 (15.0%)	
M Stage			0.242			0.717
0	56 (98.2%)	46 (93.9%)		24 (92.3%)	19 (95.0%)	
1	1 (1.8%)	3 (6.1%)		2 (7.7%)	1 (5%)	
Surgical Margin			<0.001*			<0.001*
Negative	45 (78.9%)	10 (20.4%)		23 (88.5%)	7 (35.0%)	
Positive	12 (21.1%)	39 (79.6%)		3 (11.5%)	14 (65.0%)	
Radiomics score Median, (interquartile range)	-1.04 (-2.24~ -0.20)	0.60 (-0.31~ 1.65)	<0.001*	-0.95 (-2.07~ -0.27)	0.75 (-0.71~ 2.29)	<0.001*

DN-SNSCC, de-novo SNSCC; IP-SNSCC, inverted papilloma-derived SNSCC; (*P < 0.05) .

**Figure 2:**
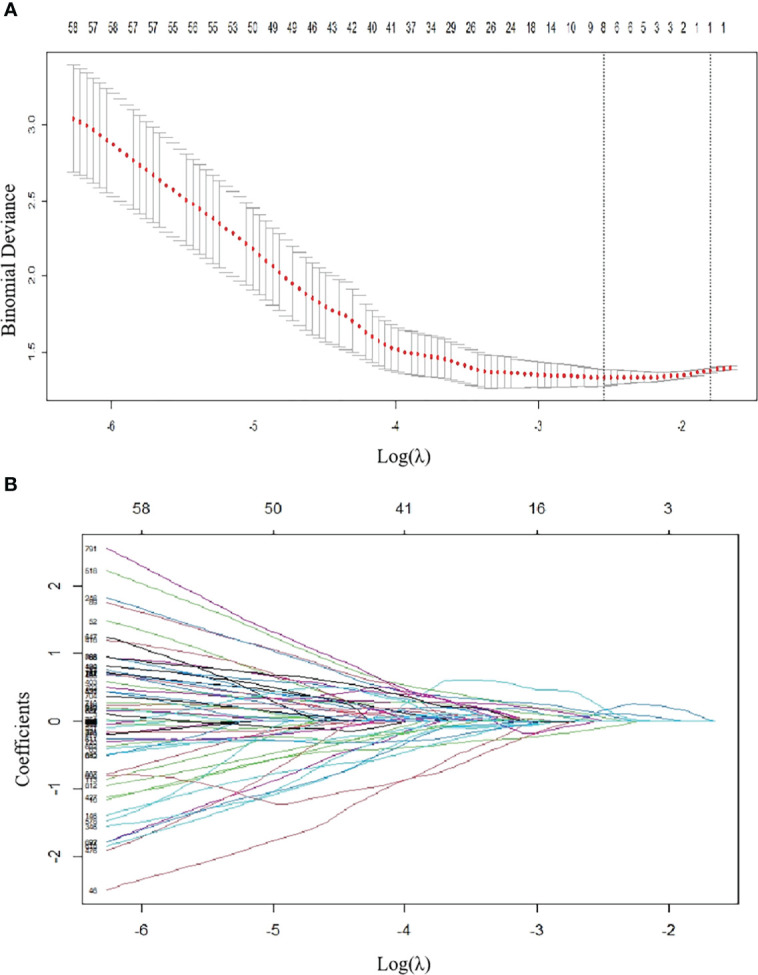
Radiomics feature selection using LASSO regression in the training group. **(A)** *Via* 7-fold cross-validation(CV), the value of λ that gave the minimum average binomial deviance was used to select features. The y-axis shows binomial deviances and the lower x-axis the log(λ). Numbers along the upper x-axis indicate the average number of predictors. Red dots indicate average deviance values for each model with a given λ, and vertical bars through the red dots indicate the upper and lower values of the deviances. By using the minimum criteria and the 1 standard error of the minimum criteria (the 1-SE criteria), the vertical black lines define the optimal λ values = 0.07873. **(B)** The coefficients have been plotted vs. log(λ). The features with nonzero coefficients are shown in the plot.

A total of 850 radiomic features were extracted from the ROIs. After the reproducibility analysis, we derived 768 features with ICC>0.75. Based on LASSO regression, these 768 features were reduced to 8 optimal features **(** **Figure 2**). Then, we used the 8 radiomic features with nonzero coefficients to construct the radscore as follows:


$$\begin{array}{cccccccc} \text{Radscore}\;=1.497\times \text{waveletHLH}\_\text{firstorder}\_\text{Skewness} & -\;0.123\times \text{waveletHHH}\_\text{gldm}\_\text{DependenceVariance}\left(\text{DV}\right) & -\;0.001\times \text{waveletLHH}\_\text{firstorder}\_90\text{Percentile} & +\;\;0.002\times \text{waveletLHL}\_\text{firstorder}\_\text{Maximum} & +\;\;0.195\times \text{waveletHLL}\_\text{firstorder}\_\text{Kurtosis} & +\;21.\;\;01\times \;\;\text{original}\_\text{gldm}\_\text{DependenceNonUniformityNormalized}\left(\text{DNUN}\right) & -\;8.176\times \text{original}\_\text{gldm}\_\text{SmallDependenceEmphasis}\left(\text{SDE}\right) & -\;\;4.\;088\;\;\times \;\text{original}\_\text{shape}\_\text{Flatness}-3.433 \end{array}$$

ER SNSCC lesions had significantly higher radscore values than NER SNSCCs in both cohorts (*P* < 0.001).

**Figure 3:**
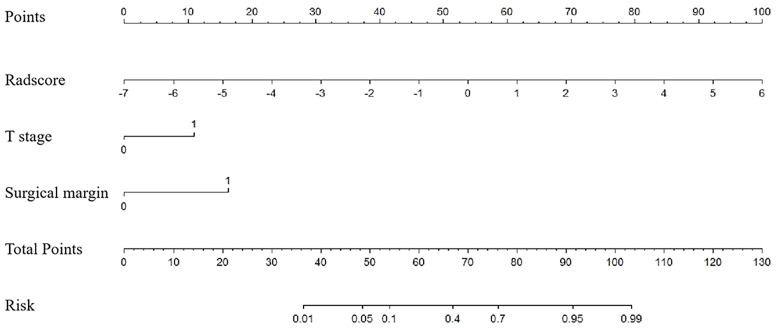
Nomogram for risk prediction of ER with the radiomics signature (Radscore) and clinical factors (T stage and surgical margin) incorporated.

Based on univariate analysis, the clinical T stage and surgical margin were found to be clinical risk factors for ER in SNSCCs. Based on logistic regression analysis, the radscore and clinical risk factors (including T-stage and surgical margin) were incorporated into the nomogram as independent predictors of ER (**Figure 3**).

**Figure 4:**
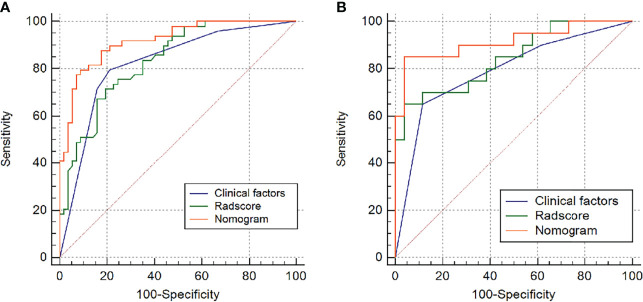
Receiver operating characteristic (ROC) curves of the radiomics model, clinical model and nomogram model in the **(A)** training group and **(B)** testing group.

**Figure 5:**
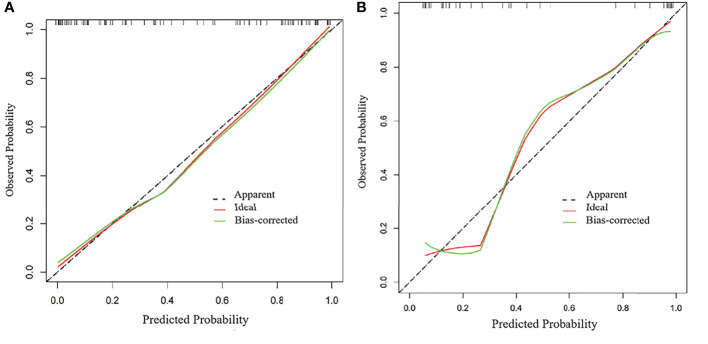
Calibration curves of the radiomics nomogram in the **(A)** training group and **(B)** testing group.

**Table 2** and **Figure 4** show the differential ability of the clinical factors (T stage and surgical margin), radscore and nomogram model to identify the differentiation grade of SNSCCs. The AUCs for the nomogram, clinical factors and radscore were 0.92, 0.82 and 0.84, respectively, in the training cohort and 0.92, 0.79 and 0.84, respectively, in the testing cohort. When the Hosmer-Lemeshow goodness-of-fit test was applied, the calibration curve of the nomogram (**Figure 5**) demonstrated very good reliability in evaluating ER in the training and testing cohorts (*P* > 0.050).

**Table 2:** AUCs of the Radscore, Clinical model and Nomogram model.

	Training cohort		Testing cohort	
	AUC (95%CI)	*P*-value	AUC (95%CI)	*P*-value
Radscore	0.84 (0.76-0.91)		0.84 (0.73-0.96)	
Clinical model	0.82 (0.75-0.90)		0.79 (0.66-0.92)	
Nomogram	0.92 (0.87-0.97)		0.92 (0.82-1.00)	
Radscore *vs.* Clinical model		0.831		0.528
Nomogram *vs.* Radscore		0.003*		0.177
Nomogram *vs.* Clinical model		0.004*		0.016*

(*P < 0.05).

**Figure 6:**
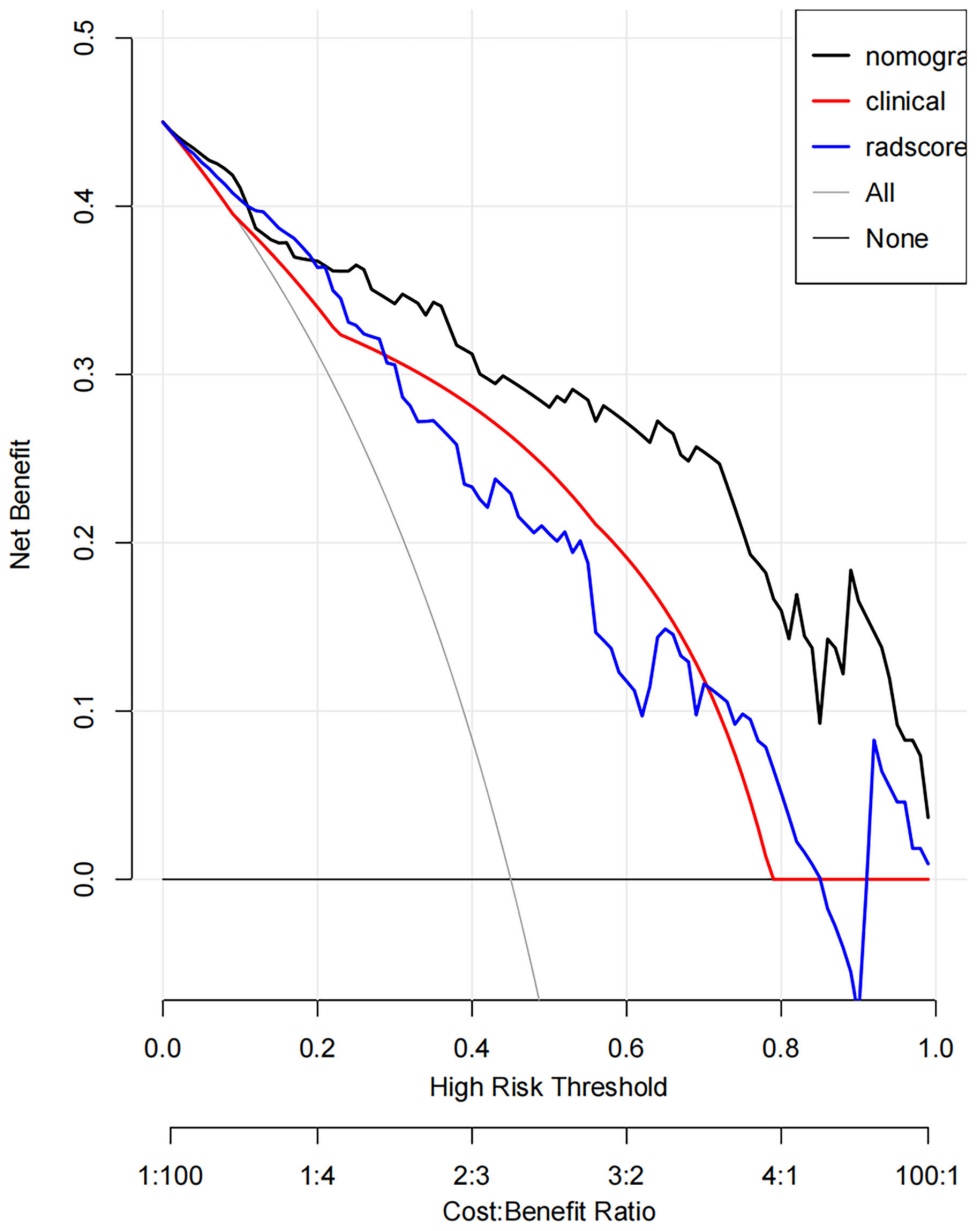
Decision curve analysis (DCA) derived from the testing cohort showed that if the threshold probability was <10% and >20%, the use of the nomogram to evaluate the grade offered more benefits than either the treat-all scheme (assuming all SNSCCs were ER) or the treat-none scheme (assuming all SNSCCs were NER).

DCA showed that if the threshold probability was 0.19-1.00, the use of the nomogram to evaluate the ER offered more benefits than either the treat-all scheme (assuming all SNSCCs were ER) or the treat-none scheme (assuming all SNSCCs were NER) (**Figure 6**). In addition, using the same threshold probability, the nomogram could add more benefits than either the strategy involving exclusive use of the radscore or the strategy involving exclusive use of clinical factors.

**Figure 7:**
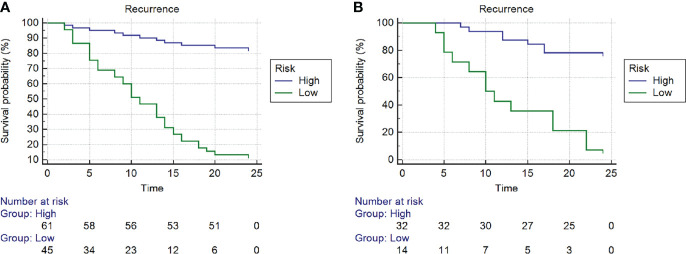
Kaplan-Meier curves of recurrence-free survival (RFS) of high- and low- risk subgroups according to the cut-off value of nomogram in the **(A)** training cohort and **(B)** training cohort.

The optimum cutoff value of the nomogram generated by the X-tile plot was -0.59 on the basis of the training cohort. Accordingly, patients were classified into the high- and low-risk subgroups. Kaplan-Meier analysis showed that in the training cohort, the 2-year RFS rates were 83.4 ± 4.8% for low-risk patients and 13.3 ± 5.1% for high-risk patients (*P* < 0.001). The training cohort showed similar results; the 2-year RFS rates were 78.1 ± 7.3% for low-risk patients and 7.1 ± 6.9% for high-risk patients (*P* < 0.001). (**Figure 7**).

## Discussion

In the present study, we developed and validated a combined nomogram model for the prediction of ER in SNSCC patients. A radscore based on eight features was useful for evaluating ER. By incorporating the radscore and clinical factors, the nomogram model achieved higher predictive value. The calibration curve DCA showed the good clinical utility of this easy-to-use nomogram prediction model.

The most common malignant sinonasal tumor is SNSCC, which comprises 50% of all cases (12). Yan et al. (4) reported that a large proportion of SNSCCs diagnosed at an advanced stage showed worse disease-free survival than early stage tumors. In addition, over ten years of follow-up, the researchers found that SNSCC recurrence occurred very early (within 3 years after resection). This finding was also supported by Quan et al. (13), who demonstrated that local relapse was the most important reason for treatment failure in SNSCC patients. Thus, early prediction of the risk of relapse is very important to implement effective individualized treatment.

Radiomics has recently become a research hotspot in oncology. By extracting high-throughput quantitative data characterization algorithms, radiomics provides unprecedented opportunity for improved machine learning powered predictive models of head and neck cancers. These models not only predict survival but also on risk of relapse (14). DWI can reflect the random movement of water molecules at the cellular level, and the apparent diffusion coefficient (ADC) derived from DWI has been shown to be a promising biomarker for characterizing tissues.

In our study, using LASSO, a total of 850 ADC-based features were narrowed to only 8 potential predictors. These 8 radiomic features were divided into 3 types: first-order, texture (GLDM) and shape features. First-order statistics are also known as intensity-based features. Histogram analysis in our study revealed higher signal skewness and kurtosis values in ER SNSCCs than in NER SNSCCs, explaining the asymmetry of the histogram distribution within tumors (15). Kierans et al. (16) indicated that ADC skewness could reflect the heterogeneity of cellular environments, resulting from a high degree of cellular atypia and nuclear pleomorphism. This information is also useful for the differentiation of high- versus low-grade carcinoma. Hirata1 et al. (17) proposed that ADC histogram-derived parameters of kurtosis were significantly correlated with RFS (*P* < 0.001) in esophageal cancer patients. In the analysis of texture-based features, a higher DV value further elucidated the high heterogeneity in the ER SNSCC group. Another significant radiomic predictor is a shape-based feature, namely, flatness. In our study, SNSCC with ER generated a more irregular shape and lower flatness value. This result is consistent with a recent study by Khodabakhshi et al. (18), which reported a significant correlation between higher values of flatness and better survival outcomes in renal cell carcinoma patients. However, these above features could only reflect one aspect of tumor information. Therefore, by integrating the eight ADC-based radiomic features regarding heterogeneity and shape of the whole tumor, the radscore achieved moderate power in discriminating ER of SNSCCs with AUCs of 0.84 in both of the training and testing cohorts.

The clinical risk factors associated with the ER of advanced SNSCC tumors are seldom addressed in previous literature. Li et al. (19) indicated that in T4 stage SNSCC patients, invasion of the orbit and brain could lead to incomplete resection of tumors and ER. This finding is in agreement with recent studies (6, 13) that reported that the higher the T stage, the greater the probability of recurrence. In addition, the relationship between a positive surgical margin with a higher local recurrence rate and poor survival outcome has been reported in several previous studies (20, 21). However, these clinical factors, which are mainly based on anatomical structures, are not accurate enough due to the highly heterogeneous tumor biology, which can significantly affect patient survival.

Nomogram is a new method to estimate prognosis by incorporating multiple relevant factors and can be readily used in clinical practice. Recent evidence (5) has indicated that a nomogram combining radiomic features and clinical characteristics could be effectively applied in evaluating the recurrence of some types of cancers, such as nasopharyngeal carcinoma, gastric cancer (6) and hepatocellular carcinoma (7). In the present study, multivariate analysis revealed that the radscore and several clinical factors, including T stage (T3 or T4) and surgical margin (negative or positive), were independent predictors of ER in advanced SNSCCs and were included as candidate factors in the nomogram. After comparison, the nomogram showed significantly greater effectiveness (AUC of 0.92) than either the radscore or clinical factors in discriminating ER of SNSCCs in the training cohort. In the testing cohort, the nomogram also tended to show the best performance in predicting ER, which suggested that the nomogram could serve as an important marker for evaluating ER and prognosis. For further study, we identified the optimal cutoff of the nomogram value and divided the patients into different categories of risks using X-tile software. Kaplan-Meier curves revealed a significant difference in RFS between the high- and low-risk SNSCC patients in both the training and testing cohorts. Thus, the nomogram has a significant impact on treatment decisions. Thus, if ER is strongly indicated by the clinical-radiomics nomogram model, clinicians may wish to consider additional or alternate treatment plans.

We also employed DCA to further quantify the clinical utility of this radiomic nomogram model, considering the clinical consequences of decisions. DCA is a new method based on the analysis of threshold probabilities to express the net benefit. In our study, DCA showed the benefit of applying the nomogram as opposed to the clinical factor model or the radscore model for individualized prediction of the probability of ER in advanced SNSCC patients.

Our study has several potential limitations. First, due to the rarity of advanced SNSCC and difficulty in collecting patient data, the number of samples was limited. Second, our study was conducted in a single institution. Multi-center studies are requisite to increase the effectiveness of the nomogram. Thirdly, it is a major challenge to select the most valuable features from high-dimensional and small-sample data; thus, other machine-learning algorithms need to be investigated in the future to yield preferable outcomes. In addition, the ADC values were derived from a monoexponential model of DWI features, whereas biexponential [e.g., intravoxel incoherent motion (IVIM)] and non-Gaussian distribution-based DWI features could yield more robust parameters to characterize tumor heterogeneity.

To our knowledge, the current study is the first report to indicate the utility of a radiomics monogram incorporating both quantitative ADC-based radiomic features and clinical factors in advanced SNSCC patients. Our preliminary study demonstrated that the nomogram represents a promising tool in the prediction of ER in SNSCC and can be conveniently applied to facilitate individualized treatment.

## Data Availability Statement

The original contributions presented in the study are included in the article/**Supplementary Material**. Further inquiries can be directed to the corresponding author.

## Ethics Statement

The studies involving human participants were reviewed and approved by Eye and ENT Hospital of Fudan University. The patients/participants provided their written informed consent to participate in this study. Written informed consent was obtained from the individual(s) for the publication of any potentially identifiable images or data included in this article.

## Author Contributions

NL, SY, and ML designed the research. NL, SY, ML, YShi, ZX, and WC collected the data. NL, SY, ML, and YShi contributed data analysis tools and performed the analysis. NL wrote the paper. YSha and YC supervised the study. All authors contributed to the article and approved the submitted version.

## Conflict of Interest

The authors declare that the research was conducted in the absence of any commercial or financial relationships that could be construed as a potential conflict of interest.

## Publisher’s Note

All claims expressed in this article are solely those of the authors and do not necessarily represent those of their affiliated organizations, or those of the publisher, the editors and the reviewers. Any product that may be evaluated in this article, or claim that may be made by its manufacturer, is not guaranteed or endorsed by the publisher.
